# Optimizing Quality of Care and Patient Safety in Malaysia: The Current Global Initiatives, Gaps and Suggested Solutions 

**DOI:** 10.5539/gjhs.v8n6p75

**Published:** 2015-10-20

**Authors:** Mu’taman Jarrar, Hamzah Abdul Rahman, Mohammad Sobri Don

**Affiliations:** 1 College of Business, Universiti Utara Malaysia, Kedah, Malaysia

**Keywords:** quality of care, patient safety, optimizing quality, medico legal complaints, staffing, work environment

## Abstract

**Background and Objective:**

Demand for health care service has significantly increased, while the quality of healthcare and patient safety has become national and international priorities. This paper aims to identify the gaps and the current initiatives for optimizing the quality of care and patient safety in Malaysia.

**Design:**

Review of the current literature. Highly cited articles were used as the basis to retrieve and review the current initiatives for optimizing the quality of care and patient safety. The country health plan of Ministry of Health (MOH) Malaysia and the MOH Malaysia Annual Reports were reviewed.

**Results:**

The MOH has set four strategies for optimizing quality and sustaining quality of life. The 10^th^ Malaysia Health Plan promotes the theme “1 Care for 1 Malaysia” in order to sustain the quality of care. Despite of these efforts, the total number of complaints received by the medico-legal section of the MOH Malaysia is increasing. The current global initiatives indicted that quality performance generally belong to three main categories: patient; staffing; and working environment related factors.

**Conclusions:**

There is no single intervention for optimizing quality of care to maintain patient safety. Multidimensional efforts and interventions are recommended in order to optimize the quality of care and patient safety in Malaysia.

## 1. Introduction

The Institute of Medicine’s (IOM) (2000) report to ‘Err Is Human’ stated that 98,000 deaths in United States occurred annually as result of medical errors (IOM, 2000). One study conducted in Australia stated that adverse events occurred for 17% of all admitted patients and were mostly considered preventable (Wilson et al., 1995). Recently, 400,000 adverse events and 210,000 deaths annually have been associated with preventable harm in the US hospitals (James, 2013). In the 15 years since the IOM Report, there have been multidisciplinary interventions and system reform to prevent patient harm. Despite all these efforts, preventable harm in the hospitals is still substantial (Leape, 2015). In European countries, there are not enough nurses in the healthcare facilities to fulfill increasing demands, which in turn is negatively associated with the quality of care and patient safety (Hinno, Partanen, & Vehviläinen-Julkunen, 2011). In Malaysia, there were increases in the demand for and cost of care coupled with the lack of resources, which in turn, threaten the sustenance of the performance of the Malaysian health system (MOH, 2011b). Additionally, the lack of money and resources is negatively associated with patient safety and the quality of care (Steiger, 2007). So, increasing demand for care and longer working hours, combined with limited budgets, have made it crucial to sustain the outcomes of care (Drake, 2013). Thus, the main purpose of the paper is to explore the gaps of optimizing the quality of care and patient safety in Malaysia.

## 2. Method

Highly cited articles were reviewed and used as the basis to retrieve and critically examine the current initiatives for optimizing the quality of care and patient safety in Malaysia. The main sources of information of this review were papers published in peer reviewed research journals. PubMed, Medline, Science Direct and Google Scholar databases were resourced extensively and used to identify the factors affecting quality of care based in the global current literature. The purpose of this paper was to identify the gaps for optimizing the quality of care and patient safety by reviewing the most recent international literature. The current initiatives and its implications in the Malaysian healthcare system were highlighted. The country health plan of Ministry of Health (MOH) Malaysia and the MOH Malaysia Annual Reports were used to identify these initiatives and compared with the global initiatives.

## 3. Results

Today, managers face challenges to ensure patient safety and to improve the quality of healthcare. There are many determinants affecting the performance of healthcare services. Staff competency level, leadership style, organizational cultures, working environment, team cohesiveness, compliance with international standards among others, all have been considered variables that affect the outcomes of hospitalized patients.

Research in quality improvement helps professionals, researchers and healthcare providers to improve the quality of care in their organizations. By review the existing literature, it is found that quality of care defined differently among the pioneers. So, this review highlights these definitions. The gaps for optimizing healthcare quality, and to identify their implications on the Malaysian healthcare system were then explored.

### 3.1 Definitions of Quality

The IOM (2000) defined quality of care as ‘‘the degree to which health services for individuals and populations increase the likelihood of desired health outcomes and are consistent with current professional knowledge’’(IOM, 2000). Quality of care is defined differently among providers, insurers and patients (Montgomery, Todorova, Baban, & Panagopoulou, 2013). Insurers and providers defined the quality of care as the effectiveness of care by using appropriate clinical guidelines and standards for patient care, whereas patients define it as effective, easily accessible, and available and includes consistent information (Campmans-Kuijpers et al., 2013). Furthermore, Wicks & Roethlein (2009) defined quality based on patient satisfaction as “the summation of the affective evaluations by each customer of each attitude object that creates customer satisfaction” (p. 83). This customization in satisfying the needs and wants of customers make the perceived healthcare service quality varied among customers. Similarly the pioneers of healthcare quality management defined the quality of care differently. For example, Joseph Juran defined it as “fitness of use” to avoid dissatisfaction of customers, (Pelletier & Beaudin, 2005) while, Philip B. Crosby defined it as “Do it right the first time” (McLaughlin & Kaluzny, 2004). Generally, one could conclude that quality of care is the excellence of care (Gillespie, 2007). The excellence of care is more subjective and varies from one individual, or perspective, to another, thus, quality of care is defined differently among various individuals or contexts. To better understand quality in healthcare organizations one must examine quality in manufacturing companies.

#### 3.1.1 Industrial vs. Healthcare Quality

Healthcare quality varies among other disciplines and healthcare institutions and the point of product type is also varied in the manufacturing industry. Manufacturing companies deliver tangible goods and any products with defects can be returned back by the customer which resolves most issues (Guo & Hariharan, 2012). On the another hand Guo & Hariharan (2012) demonstrated that healthcare organizations deliver services to their customers and the “defects” of the service causes harm and is irreversible in some circumstances. For instance, scarring, permanent loss of mobility, organ failure and death cause irreversible harm. The survival of the organization may be jeopardized by increasing number of lawsuits and loss of customers. Critical to providing defect free services when so much is at stake in patients’ lives healthcare organizations must proactively optimize the quality of healthcare they deliver. Thus, elements of improving quality of care were adopted from other industries to meet client expectations (McLaughlin & Kaluzny, 2004). Donabedian, (1993) illustrated that industrial quality approach is limited and ignores the patient-provider relationship, whereas healthcare quality requires more attention to client needs and to their expectations. Satisfying client expectations may require medical and healthcare staff to have better education and training. Donabedian also argued that industry processes are routine, require standardized input and output, which makes work flow linear and repetitive (McLaughlin & Kaluzny, 2004). On the other hand, it is impossible to guarantee standardized inputs and outputs in healthcare because the same diseases and symptoms produce variety in outputs which depend on many variables. Those variables could be patient related variables (Weingart et al., 2011; Woodard et al., 2012; Young, Sullivan, & Duan, 1999); staffing related variables (Baron, Morris, Dye, Fielding, & Goulden, 2006; Bhavsar et al., 2007; Cramer, Jones, & Hertzog, 2011; Estabrooks et al., 2009; Gajic et al., 2008; Versa & Inoue, 2011); or environmental variables (Chehab et al., 2001; Djukic, Kovner, Brewer, Fatehi, & Cline, 2013; Hearld, Alexander, Fraser, & Jiang, 2008; Nantsupawat et al., 2011). In order to reduce the variation in care outcomes and to optimize healthcare services, healthcare organizations have to deliver care “consistent with the current professional knowledge” (Lohr & Schroeder, 1990, p. 707). Delivering care consistent with the current knowledge requires compliance with current evidence based practices in order to deliver best care practice (Ballard, 2003). To reduce controllable variation, organizations must have a working knowledge of the multiple factors affecting healthcare quality based on the evidence and current practices. So, it is important to highlight the gaps of optimizing the outcomes of care in Malaysia based on the current literature.

### 3.2 Optimizing the Quality of Care and Patient Safety in Malaysia

#### 3.2.1 The Country Profile

Malaysia is an upper middle income developing country (Tan et al., 2014), has a multiracial population, consisting of Malays 67.4%, Chinese 24.6%, Indians 7.3%, and 0.7% other ethnic groups (MOH, 2011b). In 2008, Malaysia was the 19th largest trading nation in the world, with trade excess of USD 270 billion and per capita income of USD 6,726 (WHO, 2010). The incidence of poverty has been decreasing sharply over the last few decades. The incidence of poverty in 1990, 2000, 2004 and 2008 was 49.3%, 16.5%, 5.7% and 3.8%, respectively (MOH, 2011b). Thus, Malaysia is moving towards realizing the 10^th^ Malaysia Plan to become a high income nation (MOH, 2011b).

#### 3.2.2 Malaysian Healthcare System

Malaysian healthcare services are provided by the public sector, private sector and non-MOH organizations (Merican & bin Yon, 2002). MOH Malaysia is responsible for the population’s health (Merican & bin Yon, 2002). Table 1 highlights the characteristics of these sectors, according to data obtained from the official website of MOH Malaysia. The public sector is the major provider of healthcare and consists of 141 hospitals. A total of 28,949 doctors and 56,503 nurses are working in public healthcare facilities, delivering services for patients, with a capacity of 39,728 beds.

**Table 1:** The characteristics of the Malaysian healthcare sector

Characteristics	Public	Private	Non-MOH	Total
Hospitals	141	214	8	363
Beds	39,728	14,033	3,708	57,469
Doctors	28,949	11,697	6,270	46,916
Nurses	56,503	26,653	6,011	89,167
Community nurses	23,971	267	181	24,419
Dental nurses	2,706	-	87	2,793

Source: according to the data obtained from the official website of MOH Malaysia as accessed on 31 January 2015 (http://www.moh.gov.my/english.php/pages/view/405).

The private sector is the second main provider of healthcare services (Merican & bin Yon, 2002), and consists of 214 hospitals. A total of 11,697 doctors and 26,653 nurses are working in the private healthcare facilities, and delivering services for patients, with a capacity of 14,033 beds. The private healthcare facilities include private hospitals, medical clinics, hemodialysis centers, dental clinics, hospices, maternity homes, private psychiatric hospitals, ambulatory care centers, nursing homes, psychiatric nursing homes, blood banks and community mental health centers (MOH, 2011a).

The non-MOH organizations include the care delivered by the Ministry of Education, the Ministry of Human Resources, Ministry of Defense, Ministry of Rural Development, and the Ministry of Housing and Local Government (Merican & bin Yon, 2002). The non-MOH organizations complement the role of MOH with eight hospitals, having a total capacity of 3,708 beds. A total 6,270 doctors and 6,011 nurses are employed in these healthcare facilities.

**Figure 1:**
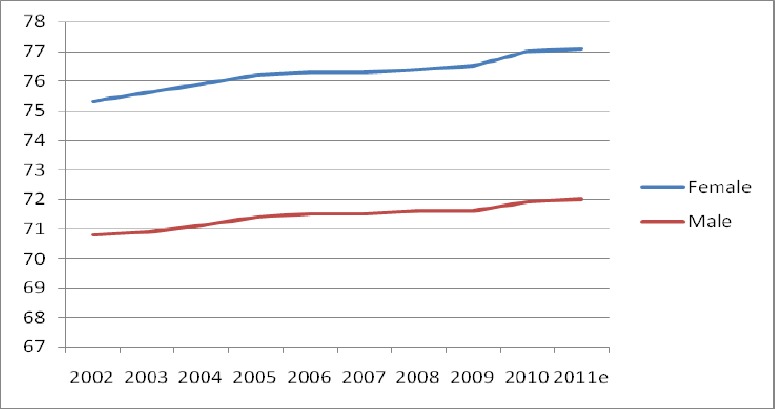
Life expectancy in Malaysia Source: Annual Report of MOH Malaysia as obtained from the Department of Statistics MOH, 2011a

The health status in Malaysia has grown, and Malaysians today live longer lives (MOH, 2011a). The estimated life expectancy at birth, based on 2010 data, has increased to 77.0 years for females, and 71.9 years for males, as compared to records in 2002, where it was 75.3 years for females, and 70.8 years for males as shown in Figure 1.

**Figure 2:**
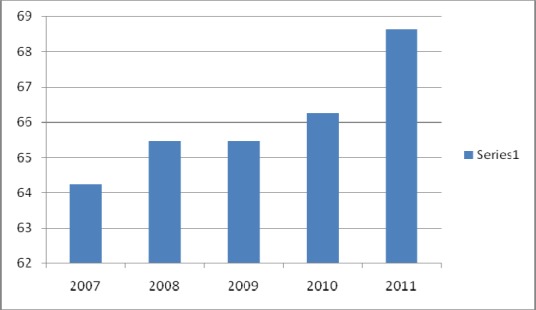
Bed occupancy rate (%) in Malaysian hospitals Source: Annual Report of the MOH Malaysia as obtained by the Health Informatics Center MOH, 2011a

The MOH has carried out regular health reforms and has implemented medical tourism to attract foreign patients in efforts to become a high income nation (Merican & bin Yon, 2002). The MOH Malaysia has the mission of facilitating and supporting the population’s health and providing high quality of care, characterized by patient-centeredness, equitable, efficient, affordable and environmentally adaptable care with emphasis on respect for human dignity (MOH, 2011a). According to the Prevention and Control of Infectious Disease Act (1988), it is mandatory to notify the state health office in order to take actions to control the spread of diseases (Aljunid et al., 2012). However, there are challenges in sustaining the quality and patient safety in Malaysia. As the population increases (MOH, 2011a), demand for healthcare increases as well (MOH, 2011b). Further, the total bed occupancy rate in the hospitals is increasing as shown in Figure 2. This increase in the demand for care and bed occupancy rate of hospitals creates challenges for the MOH in realizing its mission of delivering high quality care. Thus, the key issues of optimizing the quality of care and patient safety are highlighted in the next section.

#### 3.2.3 The Quality of Care and Patient Safety in Malaysia

Quality assurance activities have been introduced in Malaysian hospitals since 1985 in order to provide the best healthcare for the population (Reerink & Alihussein, 1990). At that time, the MOH Malaysia set quality indicators to monitor the hospitals’ performance, and found that nurses and doctors are not trained to participate in the quality improvement programs (Reerink & Alihussein, 1990). The importance of improving quality of care and patient safety in Malaysian hospitals is visible in the growing attention to reduce medical errors, waste and inefficiency in the healthcare sector (Husin, Rashid, & Othman, 2012). Further, the rapid growth in healthcare industry competition, similar to private medical centers, has led leaders to guarantee care outcomes are promptly delivered by their healthcare professionals (Husin et al., 2012).

Demographic changes in the population structure in Malaysia challenge the sustenance of the outcomes of care (John, Mani & Azizah, 2004). By 2050, it is expected that one out of every five Malaysians will be over 60 years old (John et al., 2004). The patient care outcomes of a cross-sectional study conducted in Ampang Hospital, Kuala Lumpur, found that the quality of care is better among younger patients than patients older than 40 (Priscilla et al., 2011). This shows the future challenges for optimizing the outcomes of care in Malaysia.

The Malaysian government has set an agenda to realize Vision 2020 to become a “high income nation”, by transforming healthcare, improving quality and sustaining quality of life (MOH, 2012). The MOH has set four strategies to realize these objectives: transform the healthcare system comprehensively; maintain health awareness; empower the community; and ensure universal access to healthcare for achieving the status of a high income economy (MOH, 2011b). The 10^th^ Malaysia Health Plan promotes the theme “1 Care for 1 Malaysia” in order to sustain the quality of care (MOH, 2011b). Along with these efforts, the total number of complaints received by the medico-legal section of the MOH is increasing, and the amount of compensation is sharply increasing as well (MOH, 2011a). This indicates that it is pertinent to explore factors affecting the quality of care and patient safety in Malaysia.

A study conducted in a teaching hospital in Kuala Lumpur revealed that poor communication is negatively associated with patient satisfaction and the quality of care among cancer patients (Ezat, Fuad, Hayati, Zafar, & Kiyah, 2014). Hence, dissatisfied patients are likely to complain about the perceived care ten times more than a satisfied patient (Gabbott & Hogg, 1998). Similarly, a study conducted in a Malaysian hospital found that nurse and patient communication is important in building patient trust (Maskor & Krauss, 2013). Nurses need to smile and maintain eye contact with the patient and understand the nonverbal communication to ensure patient comfort (Maskor & Krauss, 2013). In 2011, the private medical practice control section in Malaysia recorded a total of 312 patients’ and family complaints (MOH, 2011a). The cost of unresolved patients’ and family complaints in one hospital with 88,000 discharges per year was estimated to be USD 4 million (Øvretveit, 2000). The most salient complaints refer to private hospitals, with 154 records (MOH, 2011a). Thus, developing strategies for improving the delivered care and reducing harm to patients in Malaysian private hospitals are becoming more of a priority. Moreover, the degree of patient-centeredness in Malaysian private hospitals needs to be investigated.

In Malaysia, there are less than 10 institutions (public and private) awarding a degree in the medical field (Khoo & Richard, 2002). Thus, medical and nursing workforce is still deficient (Jarrar, Abdul Rahman, & Shamsudin, 2015), with a low proportion of bachelor’s degree holders (Abdul Rahman, Jarrar, & Don, 2015; Khoo & Richard, 2002). A current study in the Malaysian hospitals found that less than 10.0% of nurses are holding a bachelor’s degree, and over 90.0% are holding a diploma (Coomarasamy, Wint, & Sukumaran, 2015; Yaakup, Eng, & Shah, 2014). Thus, the impact of nurses’ education and staffing levels on the outcomes of care are questionable. Additionally, a national nursing audit conducted by the Department of Research and Quality Development under the nursing division of MOH Malaysia found that nurses working in private hospitals have lower performance than nurses working in public hospitals (MOH, 2011a). This shows the importance to focus on the Malaysian private hospitals and compare with similar initiatives.

### 3.3 The Global Initiatives of Optimizing the Quality of Care and Patient Safety

Review of the current initiatives and highly cited articles show that factors affecting healthcare quality performance generally belong to three main categories: patient related factors; staffing related factors; and working environment related factors.

Patients involved and educated about their medication have better compliance of prescribed medication. Similarly, patients receiving optimal nutrition and proper nutritional instruction and education have lower length of stay, mortality rate, readmission rate, hospital acquired infection, pressure ulcer, anemia and gastric and cardiac problems (Tappenden et al., 2013). Strengthening patient-provider relationships to increase patient involvement improves quality of care and increases patient compliance with treatment (Fischman, 2010). For instance, OpenNotes developed by Robert Wood Johnson Foundation (2010), allow patients to access their electronic medical records including their provider’s progress notes, which increases patient participation in their care process and improves patient safety (Walker, Darer, Elmore, & Delbanco, 2014). Patients’ access to their medical records through OpenNotes enhances their knowledge of their health status and improves patient provider communication which allows patients to participate in their care process. For example, patients were more likely to follow-up of their abnormal lab results (Woods et al., 2013). These positive experiences of patients help to improve patients’ care quality (Feldman, Henry, Walker, Li, & Delbanco, 2013). These findings show the importance of a patient-centered approach in healthcare organizations in order to improve the outcome of patient care (Woodard et al., 2012).

Adequate staffing improves patient care outcomes (Aiken, Clarke, & Sloane, 2002; Brooten, Youngblut, Kutcher, & Bobo, 2004; Needleman, Buerhaus, Mattke, Stewart, & Zelevinsky, 2002), and leads to higher patients’ compliance with discharge instructions (Newhouse, Himmelfarb, & Morlock, 2013), lower staff burnout, higher job satisfaction (Aiken et al., 2002), and better hospitalized patient care (Needleman et al., 2002). Mismatch between patient flow and staffing leads to increased staff workload which in turn, lowers the performance of care (Boyer et al., 2012). This shows the importance of maintaining an adequate staffing for optimizing quality of care and patient safety.

A positive clinical work environment is one which contains adequate employees, sufficient equipment, strategies for continuing education and upgrading in order to retain their employee (Baumann, 2007). Employee retention leads to improve teamwork, continuity of care and better outcomes of patients care (Baumann, 2007). While, better work environment leads to better nurse satisfaction and this leads to a higher quality of care (Nantsupawat et al., 2011). Furthermore, According to the IOM (2003), improving patient safety is transformational by transforming the work environment. This could be achieved by reinforcing the change to decrease errors and enhance patient safety (Rogers, 2006). This point of view is supported by LeBrasseur, Whissell, & Ojha (2002). They illustrate that transformational leadership and learning organizations affect continuous quality improvement programs. The transformational leadership style increases executive effectiveness, empowers employees, leads to improved quality, and controls expenses (Xirasagar, Samuels, & Stoskopf, 2005). Thus, healthcare organizations should require transformational and evidenced based leadership who can develop interdisciplinary teamwork, a learning culture and involve staff to improve quality and patient safety (Ferguson et al., 2007). In addition, adherence to external accreditation standards leads to improved quality and patient safety and enhances care process and outcomes (Scott, Poole, & Jayathissa, 2008). This also reduces patient harm mostly caused by variation in practice and noncompliance with evidence based guidelines (Ibrahim, Jeffcott, Davis, & Chadwick, 2013). In addition, compliance with standards and guidelines leads to improved clinical outcomes and lower costs for patients (Fritz, Cleland, & Brennan, 2007). These current initiatives will help to bridge the gaps of optimizing the quality of care and patient safety in Malaysia.

## 4. Discussions and Implications

Today hospital quality has become national and international priorities. Studies investigating the staffing level, shift length and work environment in Malaysian hospitals are limited. Thus, optimizing quality of care and patient safety in Malaysia, the current global initiatives and the gaps for improving quality of care and patient safety were highlighted.

To improve quality of care, patient safety and decrease care variation, evidenced based practices are required (De Lusignan, Wells, Shaw, Rowlands, & Crilly, 2005). After reviewing the current initiatives of optimizing the quality of care and patient safety, the following are the main implications and lessons learned which could help researchers, policy makers, leaders, care organizations and providers to sustain the performance of delivering best practices:

1) Patients’ demographics and clinical case complexity on the outcomes of care required to be considered in the current studies in Malaysia. Further research is required to explore the effect of these factors on the care outcomes.
2) Patients’ needs and wants are core to caring processes, so preparing skilled, trained, educated and empowered staff and maintaining healthy work environments are needed to increase patients’ satisfaction, engagement and participation in their care process.
3) There is consensus that adequate staffing is an essential component that positively improves quality of care and patient safety. Thus, hospitals need to engage in contingency planning to prevent exceeding their occupancy limits to prevent overcrowding and subsequent errors resulting from overloaded staff.
4) Inexperienced and newly appointed staff should be trained to work in interdisciplinary teams and in quality improvement in order to deliver best practices.
5) Hospitals should maintain adequate employee, sufficient equipment, strategies for continuing education, training and upgrading and improving interprofessional collaboration to maintain a positive clinical work environment to deliver best care practices.
6) Healthcare organizations should seek or maintain accreditation such as from the Magnet recognition or Pathways to Excellence programes in order to sustain a healthy work environment and care excellence and to retain and attract skilled employees.
7) Leaders of healthcare organizations should transform work environments to develop high functioning interdisciplinary teams, a learning culture, and engage all staff in order to improve quality and patient safety.
8) Hospitals must change the culture of blaming individual to that one of blaming broken systems in order to improve quality of care and patient safety. Appropriate resources can then be directed to improving their healthcare systems.

## 5. Conclusions

Several determinants such as staffing, work environments and patient-centeredness affect the quality of care and patient safety. These determinants have been defined as key factors affecting quality of care. Clinical evidence shows that the best way to optimize quality is to decrease care variation. In addition, optimizing quality of care should result in multidimensional improvement interventions. The MOH Malaysia has carried out regular health reforms and has set four strategies to become a “high income nation”. Despite of these efforts, the total number of complaints received is increasing. So, to deliver best practice, Malaysian healthcare organizations must engage in continuous multidimensional and multilevel efforts.

There is no single intervention to ensure quality improvement. Organizations should measure the performance of adherence to clinical guidelines by individuals, and should also measure the performance of ensuring the continuity of care and control patient flow which involves interprofessional and interdisciplinary groups of health care professionals and workers in order to improve quality of care. Implications for future research should be focused on examining multiple domains affecting quality of care and patient safety in Malaysia, so that a comprehensive model of sustainable quality and safety can be developed.
